# Obesity, Heart Failure, and Obesity Paradox

**Published:** 2017-01

**Authors:** Nikoo Hamzeh, Fatemeh Ghadimi, Rojin Farzaneh, Seyed Kianoosh Hosseini

**Affiliations:** Shariati Hospital, Tehran University of Medical Sciences, Tehran, Iran.

**Keywords:** *Obesity*, *Heart failure*, *Overweight*

## Abstract

The incidence and prevalence of obesity are fast increasing worldwide. Various indices have been used to measure and assess obesity. The body mass index (BMI) is the most common and practical of these indices. Overweight and obesity exert considerable adverse effects on the cardiovascular system. These effects are mediated through various neurohormonal and cytokine pathways, most of which are inflammatory mediators. Systolic and / or diastolic heart failure is more prevalent among obese and overweight individuals than among normal weight people. The concept of the “obesity paradox” has been proposed by some previously published studies, in which the prognosis of obese patients with established cardiovascular diseases, especially heart failure, is better than that of their leaner counterparts. In this review, we discuss the obesity paradox and its possible pathophysiologic mechanisms.

## Introduction

Obesity and overweight are global health concerns, with about half a billion adults and nearly 40 million children considered obese or overweight around the globe.^1^^, ^^2^ Current statistics suggest that the incidence and prevalence of obesity is rapidly rising, with a twofold increase in overweight and a threefold increase in obese individuals over the past 50 years.^3^^, ^^4^ It has also been reported that morbid obesity has increased at a faster rate than overweight and mild obesity.^5^^, ^^6^

Obesity is measured by means of various indices such as body mass index (BMI), body surface area (BSA), waist circumference (WC), waist-to-hip ratio (WHR), and waist-to-height ratio. The traditional criterion is BMI because it is an affordable and easy way of assessing body fat; however, factors such as muscle mass and ethnicity may change its relationship with body fatness. Although methods other than BMI have been shown to be more informative about body fat distribution, they are less pragmatic, more expensive, and mainly used for research purposes.^7^^, ^^8^

According to the BMI anthropometric tool, individuals with BMI < 18.5 kg/m^2^ are defined as underweight, BMI ranging from 18.5 to 24.9 kg/m^2^ normal, BMI between 25 and 29.9 kg/m^2^ overweight, and BMI > 30 kg/m^2^ obese. The American Heart Association has classified individuals with BMI between 30 and 34.9 kg/m^2^ as class I obesity, BMI between 35 and 39.9 kg/m^2^ as class II, BMI > 40 kg/m^2^ as severe or extreme obesity (class III), BMI = 50 kg/m^2^ as grade IV obesity, and BMI = 60 kg/m^2^ as grade V obesity.^9^

BMI, as a common measure of metabolic mass, is usually used to assess physiological parameters. WC mainly measures adipose tissue distribution in the body and is a marker of metabolic changes that are induced by obesity, but the association between WC and cardiovascular events is controversial.^10^^, ^^11^ It seems reasonable to combine BMI as a general obesity parameter with WC as an adipose tissue distribution discriminator to evaluate cardiovascular risk more precisely.^12^ Nevertheless, regardless of the index used to define obesity, studies suggest a greater risk of cardiovascular disease (CVD) in obese individuals.

Obesity has major adverse effects and is associated with major comorbidities such as CVD, hypertension, and metabolic abnormalities. It has also been suggested that obesity may cause greater harm than cigarette smoking, alcoholism, and poverty. Studies have also shown that obesity may replace cigarette smoking as the leading cause of preventable death in North America.^13^^, ^^14^

In the present review, we try to discuss obesity, obesity paradox, and its impact on the prognosis of obese patients with CVD.

***Molecular and Cellular Basis of Obesity***

Adipocytes are the main constituents of adipose tissue and are probably the most important element in the metabolic homeostasis of the body.^15^ They act as an endocrine as well as an immune organ by secreting the hormone “leptin”, a protein called “adiponectin”, C-reactive protein (CRP), and many different pro-inflammatory and anti-inflammatory cytokines.

Leptin affects energy metabolism and adipogenesis.^16^ Leptin levels increase in obesity and decline after weight loss. It has been suggested that leptin actions may control obesity by modulating food intake, inhibiting adipogenesis, and triggering lipolysis.^7^^, ^^17^

Although CRP is mainly produced by the liver in response to inflammation, adipose tissue can also secrete CRP. Inflammation itself and the subsequent release of cytokines such as CRP are considered to have a role in altering the cardiovascular risk state.^18^ Studies have shown that levels of CRP and other inflammatory cytokines may affect leptin resistance and hyperleptinemia.^19^

Adiponectin, mostly produced by adipocytes, decreases with increasing obesity and is significantly low in patients with metabolic syndrome and CVD compared to healthy individuals matched by BMI. It has been suggested that chronic inflammation and the subsequent increased levels of cytokines, correlated with obesity and CVD, inhibits the production of adiponectin and leads to the perpetuation of inflammation. However, adiponectin levels are not necessarily elevated in inflammatory status not related to the diseases associated with obesity.^20^

Adipocytes play a crucial role and as such can be efficiently considered as a fuel source or inefficiently as an energy storage site but effective in inflammatory processes.^7^

***Impact of Obesity on Heart Failure***

The scientific community appears to have reached a consensus on the issue of obesity: it is certainly a major risk factor for CVD. Obesity is associated with structural and functional changes in the heart and has adverse effects on hemodynamics and left ventricular (LV) structure and function. It is logically assumed that obesity leads to an increase in the incidence and prevalence of heart failure (HF).^21^

Obesity leads to an increase in total blood volume, stroke volume, and cardiac output; nonetheless, it is correlated with a reduction in systemic vascular resistance. These are considered as an adaptation mechanisms to maintain homeostasis. Augmentation in stroke volume and the resulting increase in the cardiac output is mostly due to the increase in circulating blood volume. Although less commonly the increase in the sympathetic activation may slightly increase the heart rate, the heart rate usually does not change significantly.^22^^, ^^23^

A left shift in the Frank-Starling curve in obese persons occurs due to an elevation in blood volume and preload, resulting in consequent LV remodeling-including LV dilation and LV hypertrophy. Furthermore, left atrial enlargement is frequently seen in consequence of not only an increase in blood volume but also changes in LV diastolic filling. These all contribute to diastolic and systolic dysfunctions and eventually HF.^22^^-^^25^

The Framingham heart study suggested that there was a 5% increase in men and 7% increase in women for the risk of developing HF per unit of BMI.^26^ The duration of morbid obesity had an impact on the prevalence of HF, with a prevalence of 70% and 90% after 20 and 30 years, respectively.^27^

Metabolic alternations as well as hemodynamic changes caused by obesity may affect the incidence of HF in obese patients.^28^

***Obesity Paradox***

As noted previously, obesity is deemed an independent risk factor for developing chronic heart disease, which may eventually lead to HF. Even though increased BMI and obesity put patients at risk of CVD, recent studies have suggested that there may be an inverse relationship between obesity and the prognosis of established CVD-a phenomena known as “the obesity paradox”. There are many studies suggesting that HF patients who are obese have a better outcome than their leaner counterparts. 

Horwich and colleagues^29^ first showed that the best prognosis in HF was in overweight followed by obese patients and the prognosis was worst in underweight followed by normal BMI patients. In a cohort study of 7767 outpatients with HF , categorized by using the BMI scale, those with higher BMI (overweight and obese patients) had low crude and adjusted risks of all-cause mortality and death due to HF when compared with those with lower BMI.^30^ A retrospective review of 2707 patients with HF revealed a paradoxically improved 3-year survival rates for patients with higher BMI.^31^ Another study on more than 100000 patients with decompensated HF showed that every 5 unit increase in BMI was associated with 10% lower mortality.^32^ A randomized controlled trial of 7599 symptomatic patients with HF alongside preserved or reduced systolic function demonstrated that underweight and normal BMI patients had high mortality compared with overweight and obese participants.^33^ A meta-analysis of 9 observational studies with 28209 patients with HF showed that overweight and obese patients had reductions in cardiovascular and all-cause mortality compared with normal BMI patients.^34^

A systematic review of 6 studies demonstrated that the risk for hospitalization and total and CVD mortality was high in underweight patients with chronic HF compared to the risk for CVD mortality and hospitalization in overweight subjects.^35^

Recently, the impact of cardiorespiratory fitness (CRF) levels on the prognosis in HF has been demonstrated by several studies. These findings have suggested that higher CRF levels significantly modifies the relationship between obesity and better outcomes in HF, attenuating the obesity paradox. It is important to note that assessing higher levels of CRF for obese patients can be challenging. Nevertheless, patients with higher CRF levels are expected to have better outcomes from the same BMI category.^23^^, ^^36^^-^^41^

***Mechanisms of the Obesity Paradox***

The exact mechanism for findings in favor of the obesity paradox is unclear. There are hypotheses trying to explain some issues in this field, most of which dealing with HF as the final common pathway in most CVD. Malnutrition/inflammation complex syndrome (MICS) and endotoxin/lipopolysaccharide hypothesis may explain the obesity paradox and the inverse relationship. 

In the MICS explanation, it is believed that cardiac cachexia is an independent risk factor of mortality in patients with HF.^42^ Hypoalbuminemia and an increase in tumor necrosis factor are also seen in cardiac cachexia.^43^ The consequent inflammation is considered to be the link between cachexia and congestive HF. Cytokine activation either contributes to protein/energy malnutrition (PEM) in the settings of hypoalbuminemia or may independently lead to cachexia and PEM and, therefore, increase the mortality rate of patients with congestive HF.^44^

Endotoxin/lipid hypothesis suggests that lipids in circulatory blood bind to endotoxins and inhibit their harmful effects. Increased levels of cholesterol and lipids or hyperlipidemia provide more molecules for binding to endotoxins and remove them from circulation and prevent the subsequent inflammatory response.^44^ This theory may explain the role of lipids in neutralizing the harmful effects of endotoxins in obese patients with high lipid levels. 

Another theory explains that knowing HF is a catabolic state, obese patients have more metabolic reserve and, therefore, a more favorable prognosis, but muscle wasting in leaner patients leads to poorer outcomes.^45^^, ^^46^

Studies on neurohormonal activities are on progression and may partially explain the inverse relationship between obesity and mortality in patients with HF. The expression of circulating natriuretic peptides is reduced in overweight and obese patients.^47^^, ^^48^ It leads the obese patients to become symptomatic and present at earlier stages of HF.^21^

Although there is an augmented activation of sympathetic and renin–angiotensin–aldosterone system (RAAS) in chronic HF,^49^^, ^^50^ response to RAAS system may be attenuated in obese patients, leading to a better prognosis.^21^ Because of higher levels of blood pressure in obese patients with HF, these patients have better tolerance for drugs such as beta-blockers, RAAS inhibitors, and aldosterone antagonists at higher doses, which theoretically will cause an improved outcome in this group of patients.^50^

A recent study has shown lower sympathetic activation and lower norepinephrine levels in obese patients with chronic HF than in non-obese patients with HF. Considering that increased sympathetic activity is correlated with poorer clinical outcomes in patients with HF, these data may partially explain the mechanism of the obesity paradox in HF. The rational use of beta-adrenergic blockers in patients with HF for improving the prognosis and progression of the disease is also supported by this finding.^51^ More research about the role of norepinephrine and sympathetic activation in obese patients with HF will shed more light on this issue. 

The concept of the obesity paradox in HF has been debated by several studies. It has been postulated that studies showing the obesity paradox ignore the appreciable varieties in demographic and clinical characteristics of obese and non-obese patients with HF. Not surprisingly, obese patients with HF are younger, making age the most important confounder in some studies. Other confounders such as comorbidities should also be taken into account. Using BMI as a common measure of obesity and neglecting more accurate tools is another defect in studies suggested by some authorities.^10^^, ^^52^^-^^55^

***Impact of Weight Reduction***

Although intentional weight loss is considered to have a great impact on cardiac structural and physiological changes caused by obesity, there have been different recommendations regarding weight loss for patients with HF.^21^ Studies on weight loss in different grades of obesity in patients with HF have yielded variable results. Considering these results and the obesity paradox, major HF societies have different recommendations for weight loss in this group of patients. Some of these societies recommend weight loss for HF patients with BMI > 40 kg/m^2^, some with BMI > 35 kg/m^2^, and some with BMI > 30 kg/m^2^. None of these major societies recommends weight reduction for overweight patients with HF.^56^

However, it remains unclear whether weight loss has a positive or negative effect on the prognosis of obese patients with HF. Studies have suggested that long-term weight loss may have an adverse effect on the prognosis of patients with HF, while subjects with CVD who lose body fat rather than lean body mass may have a better prognosis.^29^^, ^^57^^, ^^58^

## Conclusion

The prevalence of overweight and obesity is on the rise worldwide. There is compelling evidence supporting the adverse impact of obesity on cardiac performance and morphology leading to HF. Nonetheless, there is also evidence supporting the existence of the obesity paradox, according to which obese patients with HF have a better prognosis than their leaner counterparts. Consensus has yet to emerge as to whether the obesity paradox is a true phenomenon. Many theories try to explain the mechanism of the obesity paradox, but they have been partially successful. The role of weight reduction in prognosis has not been fully explained, and there is no clear guideline recommendation. Further research is mandated in this field, especially as regards the role of the autonomic nervous system, to explain the possible mechanisms of the obesity paradox in HF. 

## Notes:

***This paper should be cited as:*** Hamzeh N, Ghadimi F, Farzaneh R, Hosseini SK. Obesity, Heart Failure, and Obesity Paradox. J Teh Univ Heart Ctr 2017;12(1):1-5.
